# Reproductive toxicology of environmental endocrine-disrupting chemicals in women: a cohort study protocol

**DOI:** 10.3389/fcell.2024.1335028

**Published:** 2024-06-03

**Authors:** Shuyi Zhang, Fumei Gao, Min Fu, Qiuxiang Zhang, Jing Guan, Huan Shen

**Affiliations:** Reproductive Medical Center, Department of Obstetrics and Gynecology, Peking University People’s Hospital, Beijing, China

**Keywords:** environmental disrupting chemicals, reproductive health, cohort study, per- and polyfluoroalkyl substances, organophosphorus flame retardants

## Abstract

**Introduction:** Epidemiological evidence over the last few decades has consistently shown that exposure to endocrine-disrupting chemicals (EDCs) is associated with adverse reproductive health outcomes, including male and female infertility, poor-pregnancy outcomes, and increased risk of diseases in childhood and beyond. To investigate the effects of EDCs and lifestyle on all aspects of reproduction (including early oocyte development, fertilization, embryo development, embryo implantation, abortion, and preterm birth).

**Methods:** We performed this cohort study on patients receiving *in vitro* fertilization (IVF) treatment. Biological samples including urine, serum, follicular fluid, semen, fetal tissue, decidua, and placenta, were obtained.

**Results:** By studying the correlations between reproductive outcomes and environmental pollutant exposure and lifestyle, we determined the toxicological mechanisms and health effects of EDCs on female reproductive health. We found that higher concentrations of per- and polyfluoroalkyl substances were correlated with polycystic ovary syndrome (PCOS). Using specific biomarkers, we also detected the concentrations of organophosphorus flame retardants (OPFRs) in urine and found that OPFRs may disrupt hormone homeostasis.

**Discussion:** All of these results reveal EDCs may disrupt female reproduction.

## Introduction

According to the World Health Organization, about 15% of women of childbearing age worldwide suffer from infertility (Huang et al., 2024), and by the end of the 21st century, infertility will become the third major class of disease in the world following cancer and cardiovascular disease (Attina et al., 2016). The prevalence of infertility among women of childbearing age in China is 15%–20% (Gao et al., 2022). Epidemiological evidence over the last few decades has consistently shown that endocrine-disrupting chemicals (EDCs) are associated with adverse reproductive health outcomes, including male and female infertility, poor pregnancy outcomes, and increased risk of diseases in childhood and beyond (Harley et al., 2010; Bergman et al., 2013; Messerlian et al., 2016). The Environment and Reproductive Health (EARTH) study enrolled couples undergoing *in vitro* fertilization (IVF)/embryo transfer (ET) treatment to explore the effects of environmental chemicals and lifestyle factors (such as diet and smoking) (Messerlian et al., 2018). In the 21st century, Chinese scholars focus on the influence of environmental factors and social, psychological, and biological factors on the reproductive outcomes of parents and offspring during pregnancy. However, current studies have several limitations, such as small samples and inconsistent results. To resolve these problems, we designed a cohort study with a larger sample in 2018. This cohort study enrolled couples receiving *in vitro* fertilization–embryo transfer (IVF-ET) treatment to explore all aspects of human reproduction (including sperm quality, oocyte quality, fertilization, embryo development, embryo implantation, abortion, and preterm birth). Biological samples including urine, serum, follicular fluid, semen, fetal tissue, decidua, and placenta were obtained. Follicular fluid, as the fluid human oocytes are directly exposed to *in vivo*, was shown to be an important research medium for studying the effects of EDCs on the quality and development of oocytes (Basuino and Silveira, 2016). Therefore, it is better to study the concentrations of EDCs in follicular fluid than in blood to determine their effects on oocytes and female reproductive outcomes overall.

### Study design

In 2018, we established the cohort study Peking University Environmental Reproductive Health Cohort (PKU-ERC), an ongoing perspective cohort of couples seeking care at the Reproductive Medical Center, Department of Obstetrics and Gynecology, Peking University Peoples’ Hospital, Beijing, China. Clinical physicians from the Reproductive Medical Center identified potentially eligible patients in clinical practice. Women aged 18–40 years who came to the reproductive center for their first IVF/ICSI treatment were recruited for the cohort study. Patients who received donor egg transplantation and hormone drug treatment 3 months before their first visit were excluded. Informed consent was provided by all participants who agreed to participate in the study. This study was approved by the Ethics Committee of Peking University People’s Hospital.

### Sample size

The study started in January 2018 and has recruited 1,883 couples through December 2022.

## Methods

### Self-administered questionnaires

**FIGURE 1:**
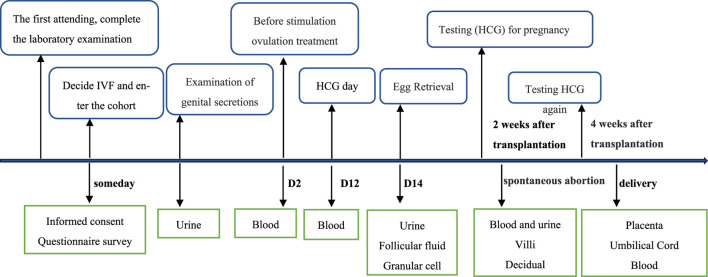
Timeline of sample collection from female individuals.

**FIGURE 2:**
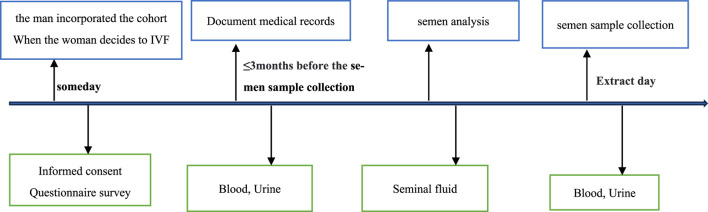
Timeline of sample collection from male individuals.

The basic personal information and samples of all enrolled patients at different stages were collected according to the established follow-up timeline (Figures 1, 2). After the patients were enrolled, the basic personal information about the enrolled couples was collected through questionnaires. First, patients provided personal information, including age, height, weight, occupation, education background, daily medication, personal and family member health, physical labor and exercise, and female reproductive history. The living environment and workplace environment, mainly including the time of exposure to decorations and their materials, ventilation time, and indoor exposure time, were also included in the questionnaire. In addition, the lifestyle questionnaires cover all aspects of lifestyle according to the Kadoorie Study of Chronic Disease in China (KSCDC) (Chen et al., 2005), including drinking, smoking, and coffee consumption. We surveyed the habits of personal and family skin products, cleaning agents, and other chemicals, such as lotions, soaps, house cleaning chemicals, plastics, pesticides, and weight-loss products. Patient depression and anxiety were also assessed using a psychiatric questionnaire.

### Biological samples

The female specimens included blood, urine, follicular fluid, villi from miscarriages, umbilical cord blood, and placenta. Male specimens included blood, urine, and semen. Women provided two fasting blood samples (on day 2 of the menstruation within 3 months before ovulation induction treatment and 3–9 days after ovulation induction). Women also provided fasting urine in the non-menstrual period within 3 months before ovulation induction therapy. For men, a fasting blood sample and fasting urine were collected before IVF/ICSI treatment. The urine, seminal fluid, and follicular fluid samples were labeled and stored at −80°C. If a woman was diagnosed with miscarriage during the fertility treatment, villi and decidua samples were collected. Placental and umbilical cord blood were collected during delivery. The umbilical cord blood was labeled and stored at −80°C. The villi, decidua, and placental tissue were labeled and stored in liquid nitrogen.

### Follow-up

In the IVF-ET treatment, the enrolled population was followed up continuously to the preset time points (Figures 1, 2). Information on the number of oocytes, the quality of the oocytes obtained, the fertilization rate, the number of high-quality embryos, embryonic development, and the number of available embryos was recorded after oocyte retrieval. After the embryo transfer, we checked the pregnancy status, which included a routine β-hCG blood test on day 14 after embryo transfer.

Biochemical pregnancy was defined by a positive pregnancy test (β-hCG). Clinical pregnancy was defined as a pregnancy confirmed by ultrasound visualization of the gestational sac 4 weeks after embryo transfer. Miscarriage was defined as pregnancy loss within 28 weeks of gestation. Live birth was defined as a baby born after 28 weeks of gestation. The neonatal weight, length, mode of delivery, and complications were recorded at delivery.

### Quantification and quality control

For the quantification of per- and polyfluoroalkyl substances, we used LC-MS/MS (Thermo Fisher Scientific, San Jose, CA, U.S.A.) as described by Lindh et al. (Lindh et al., 2012). For the quantification of organophosphorus flame retardants, urine sample extraction and analysis of 4 target metabolites (4-hydroxyphenyl diphenyl phosphate, 2-ethyl-5-hydroxyhexyl diphenyl phosphate, phenyl diptolyl phosphate, and 2-ethylhexyl diphenyl phosphate) were performed following the methods developed in our previous study (Zhao et al., 2019). All ultrasound scans were performed by experienced senior physician using a Voluson E8 (GE Medical Systems, USA). Serum hormone levels were measured by automatic chemiluminescence method using ADVIA Centaur XP immunoassay system of Siemens, Germany.

### Statistical analysis

The geometric means (GMs) and distribution percentiles for the urinary concentrations were calculated for all analytes. The correlations between the urinary concentrations of the polyfluoroalkyl substance (PFASs) or aryl-organophosphate ester (OPE) metabolites were calculated as Pearson’s correlation (r). The change in each reproductive hormone concentration was calculated as the difference in the marginal mean from the lowest quartile (Q1) to the highest quartile (Q4) divided by the marginal mean from Q1. The data analysis was performed using SPSS, version 26.0 (IBM Corporation). The statistical significance level of trends was *p* < 0.05 (Gao et al., 2022).

## Clinical data

### Biometric data

The height, weight, waist circumference, and blood pressure from the electronic records of each participant were recorded by trained study staff. According to the patient medical history, clinicians diagnosed the etiology of the disease. The ovarian reserve function and specific treatment plan of the patient were recorded in the electronic medical records.

### Laboratory analyses

The anti-Müllerian hormone (AMH), follicle-stimulating hormone (FSH), luteinizing hormone (LH), estradiol (E2), testosterone (T), and progesterone (P) levels were measured on day 2 of the treatment. The sperm quality and routine hematuria test results were as follows: free thyroxine T4, free thyroxine T3, thyroid-stimulating hormone, thyroxine peroxidase, and urine creatinine; and glomerular filtration rate. Data extracted from the electronic medical records were also recorded in the cohort file. During each fertility treatment cycle, we extracted clinical information from the electronic medical records, including the mode of conception, cycle cancellation, oocyte parameters, early embryo development, implantation, biochemical pregnancy (with β-hCG measurements), clinical pregnancy (with ultrasound assessment), and infertility diagnosis assigned by a physician. We continued to follow up those achieving pregnancy, including their pregnancy complications and pathology, glucose tolerance tests during pregnancy, and delivery outcomes (e.g., livebirths, stillbirths, birthweight, gestational age, infant sex, complications, and pathologies).

### Imaging examinations

All patients were subjected to transvaginal ultrasound examination after 2–4 days of menstruation (except for amenorrhea) using a color Doppler ultrasound diagnostic instrument with a cavity volume probe frequency of 5–9 MHz (both two-dimensional and three-dimensional scanning functions). Before the examination, the patients were asked to empty their bladder, lie flat, and flex their legs. A probe with a sterile condom was placed into the vagina, and two-dimensional ultrasound was routinely performed to observe the structure of bilateral ovaries and determine the number of antral follicle counts (AFCs) that had a diameter of 2–9 mm in both ovaries (Hao et al., 2023).

### Key findings

We detected concentrations of per- and polyfluoroalkyl substances in follicular fluid to explore their adverse effects on the reproductive health of women. We tested the concentrations of 27 perfluorinated compounds in follicular fluid. Detailed information on the demographic and clinical characteristics of the participants is presented in Table 1. The levels of AMH and LH significantly increased with increasing concentrations of per- and polyfluoroalkyl substances (Table 2). AFCs increased with the concentrations of per- and polyfluoroalkyl substances. Regression analyses found a concentration-dependent positive correlation between their concentrations and the incidence of PCOS.

**TABLE 1:** Demographic and clinical characteristics of 880 female participants in this study.

	Mean ± SD	N (%)
Age	32.32 ± 3.85	
Body mass index (BMI) (kg/m^2^)	22.99 ± 3.72	
Underweight (<18.5)	17.75 ± 0.70	
Normal (18.5∼<24.0)	21.31 ± 1.50	
Overweight (24.0∼<28.0)	25.65 ± 1.13	
Obese (≥28.0)	30.94 ± 2.54	
No. smoking		871 (99.00%)
No. drinking		879 (99.90%)
Culture		
High school and below		250 (28.41%)
College and undergraduate		529 (60.11%)
Postgraduate or above		101 (11.48%)
Reproductive information		
Infertility years	3.54 ± 2.81	
Infertility results		
Female		523 (59.43%)
Pelvic and tubal factors		406 (46.14%)
Ovulation dysfunction		81 (9.20%)
Hypoovarianism		36 (4.09%)
Male		133 (15.11%)
Both		182 (20.68%)
Unknown		42 (4.77%)
Polycystic ovary syndrome (PCOS)		149 (16.93%)
Follicle-stimulating hormone (FSH)	8.07 ± 3.13	
Luteinizing hormone (LH)	4.65 ± 2.91	
Estradiol (E2)	47.17 ± 23.72	
Progesterone (P)	0.84 ± 0.67	
Testosterone (T)	1.81 ± 0.90	
Anti-Müllerian hormone (AMH)	3.91 ± 3.22	
Antral follicle count (AFC)	11.38 ± 7.26	

**TABLE 2:** Reproductive hormone levels and antral follicle counts (AFCs) by quartile of polyfluoroalkyl substance (PFAS) concentrations among 880 women.

	Anti-Müllerian hormone (AMH)		Luteinizing hormone (LH)		Antral follicle count (AFC)	
	Adjusted odds ratio (OR) (95% confidence interval [CI])	Adjusted *p*-value	Adjusted OR (95% CI)	Adjusted *p*-value	Adjusted OR (95% CI)	Adjusted *p*-value
ΣPFAS						
Q1	Ref.		Ref.		Ref.	
Q2	0.41 (−0.18, 1)	0.169	0.12 (0.01, 0.24)	0.041	1.05 (0.99, 1.11)	0.109
Q3	0.46 (0.14, 1.05)	0.134	0.15 (0.03, 0.28)	0.013	1.11 (1.05, 1.17)	<0.001
Q4	0.91 (0.31, 1.50)	0.003	0.16 (0.05, 0.27)	0.004	1.12 (1.06, 1.19)	<0.001
*P*		0.002		0.007		<0.001

ΣPFAS is the sum of all PFAS concentrations; covariates were female age, female body mass index (BMI), female literacy, infertility years, and indications (female, nonfemale).

We measured organophosphorus flame retardants (OPFRs) in the urine of 913 of 1,286 eligible participants. Except three hydroxylated metabolites of 2-ethylhexyl diphenyl phosphate (EHDPP), all aryl-OPE–triphenyl phosphate (TPhP), tricresyl phosphate (TCrP), and the diester compound diphenyl phosphate (DPhP) were detected in the urine samples. The target metabolites 5-OH-EHDPP, 4-OH-MDTP, DPhP, and 4-OH-TPhP were detected in the urine samples at frequencies of 94.6% (864/913), 93.3% (852/913), 92.1% (841/913), and 84.2% (769/913), respectively. The reproductive hormone levels were reported as quartiles of urinary aryl-organophosphate ester metabolite concentrations according to previous studies for relevant results (Gao et al., 2022). The results showed that the quartile of 4-hydroxyphenyl diphenyl phosphate (4-OH-TPhP) was positively associated with the progesterone (P) level (*p* trend = 0.008), and the P level in the highest quartile of 2-ethyl-5-hydroxyhexyl diphenyl phosphate (5-OH-EHDPP) was 7.2% (95% CI, 5.7%–8.7%) greater than that in the lowest quartile (Gao et al., 2022). The 17β-estradiol levels in the highest quartiles of 4-OH-TPhP and 5-OH-EHDPP were 15.0% (95% CI, 13.7%–16.1%) and 5.9% (95% CI, 15.7%–16.1%) lower than those in the lowest quartile, respectively. The anti-Müllerian hormone level linearly increased across the phenyl diptolyl-phosphate (4-OH-MDTP) quartiles (*p* trend = 0.036), and the follicle-stimulating hormone level exhibited the opposite trend (*p* trend = 0.0047). These results indicate that aryl-OPEs may disrupt hormone homeostasis, according to their specific biomarkers, and may negatively affect female reproduction.

## Discussion

To explore the influence of environmental factors on infertility, this study collected biological specimens (blood, urine, follicle fluid, serum, etc.); case reports; environmental exposure factor reports; lifestyle, diet, exercise, and mental health questionnaires from the enrolled patients; and follow-up data on ovulation induction, pregnancy outcomes, and delivery outcomes. By studying the correlations between the reproductive outcomes and exposure to environmental pollutants and lifestyle, the reproductive toxicology mechanism and health effects of environmental endocrine disruptors were revealed, laying a theoretical and technical basis for the systematic evaluation of the effects of endocrine-disrupting chemicals on female reproductive health.

Per- and polyfluoroalkyl substances and organophosphorus flame retardants are endocrine-disrupting chemicals that can disturb normal reproductive outcomes and cause adverse pregnancy outcomes. Per- and polyfluoroalkyl substances refer to hydrogen atoms on one or more carbon chains that are partially or completely depleted by fluorine atoms. PFASs are a class of substituted compounds with strong thermal stability, chemical stability, and excellent surface activity (Ding et al., 2020). Therefore, they are widely used in industrial production, packaging materials, fire foam, metal plating, and pesticides (Ding et al., 2020). PCOS is a heterogeneous disorder characterized by unclear etiopathogenesis that likely involves genetic and environmental components that synergistically contribute to its phenotypic expression (Xu et al., 2024). Its main manifestations include reproductive dysfunction characterized by abnormal follicular development and hyperactivity of steroid hormone synthesis and glucose metabolism disorders characterized by insulin resistance or associated obesity and hyperinsulinemia (Xu et al., 2024). For women of childbearing age, the main manifestation is being affected by continuous anovulation or nonovulation. Evidence from animal experiments shows that PFOS exposure inhibits the maturation and ovulation of mouse oocytes (Feng et al., 2015; Wang et al., 2018). A case–control study in China (n = 367) found that the serum concentration of perfluorododecanoic acid (PFDoDA) was significantly associated with PCOS, while the serum concentration of PFOS, PFOA, and other per- and polyfluoroalkyl substances was not associated with PCOS (Wang et al., 2019). Polycystic ovary syndrome has been associated with polymorphisms caused by peroxisome proliferator-activated receptor γ (PPARγ) (Y and Valsala Gopalakrishnan, 2020). The PPARγ agonist valproic acid (an antiepileptic drug) can cause symptoms associated with polycystic ovary syndrome (Cutia and Christian-Hinman, 2023). PFHxS, PFOS, PFOA, PFNA, and PFDA are agonists of PPARγ (Li et al., 2020). Human exposure to PFAS may affect female ovarian development and lead to female infertility. PPARγ activation downregulates aromatase, the key enzyme for E2 synthesis in human granulosa cells, and upregulates the expression of 3β-HSD I mRNA (the key enzyme for progesterone biosynthesis) in theca and granulosa cells to promote progesterone secretion (Hiromori et al., 2016). Our other study found that the E2 concentration is positively correlated with increasing concentrations of 4-OH-TPhP and 5-OH-EHDPP. *In vitro* studies have found that TPhP and EHDPP can also activate PPARγ, and the PPARγ agonistic activity may explain the association with E2 levels for TPhP and EHDPP. Moreover, EHDPP elicited stronger PPARγ agonistic activity (2.04 μM) than TPhP did (2.78 μM) (Hu et al., 2017; Yao et al., 2023). PPARγ also plays an essential role in hormone synthesis in granulosa cells and the placenta (Yoon et al., 2020).

This study has several strengths. First, it had a relatively large sample and data on endpoints. Second, follicular fluid are human oocytes directly exposed, were collected. The concentration of the target organ is more likely to represent the actual level of PFAS entering the female reproductive system. This study had a detailed design, included registered couples with complete information, and controlled for potential confounders. Its main limitation is that it was conducted in women undergoing IVF. Whether the results can be generalized to all women needs to be carefully evaluated.

## Data Availability

The data of this study are not openly available and are available from the corresponding author upon reasonable request. Requests to access these datasets should be directed to HS, rmivf@sina.com.
